# A hereditary spastic paraplegia mutation in kinesin-1A/KIF5A disrupts neurofilament transport

**DOI:** 10.1186/1750-1326-5-52

**Published:** 2010-11-18

**Authors:** Lina Wang, Anthony Brown

**Affiliations:** 1 Center for Molecular Neurobiology and Department of Neuroscience, The Ohio State University, Columbus, OH 43210, USA

## Abstract

**Background:**

Hereditary spastic paraplegias are a group of neurological disorders characterized by progressive distal degeneration of the longest ascending and descending axons in the spinal cord, leading to lower limb spasticity and weakness. One of the dominantly inherited forms of this disease (spastic gait type 10, or SPG10) is caused by point mutations in kinesin-1A (also known as KIF5A), which is thought to be an anterograde motor for neurofilaments.

**Results:**

We investigated the effect of an SPG10 mutation in kinesin-1A (N256S-kinesin-1A) on neurofilament transport in cultured mouse cortical neurons using live-cell fluorescent imaging. N256S-kinesin-1A decreased both anterograde and retrograde neurofilament transport flux by decreasing the frequency of anterograde and retrograde movements. Anterograde velocity was not affected, whereas retrograde velocity actually increased.

**Conclusions:**

These data reveal subtle complexities to the functional interdependence of the anterograde and retrograde neurofilament motors and they also raise the possibility that anterograde and retrograde neurofilament transport may be disrupted in patients with SPG10.

## Background

Hereditary spastic paraplegias are a group of neurological disorders characterized by progressively increasing lower-extremity weakness and spasticity [1]. The primary cause appears to be distal degeneration of the longest ascending and descending axons in the spinal cord, though the explanation for this selective vulnerability is not known. To date, at least 41 spastic paraplegia gene loci have been mapped (termed SPG1 through SPG 41) and 17 genes have been identified [2]. The inheritance can be autosomal dominant, autosomal recessive, or X-linked. One of the autosomal dominant forms, SPG10, is caused by mutations in kinesin-1A, also known as KIF5A, which is a member of the kinesin-1 family of motor proteins.

Kinesin-1 motor proteins are heterotetramers composed of two heavy chains and two light chains [3]. There are three kinesin-1 heavy chain genes in mammals: kinesin-1A, B and C (also known as KIF5A, B and C) [4]. Kinesin-1A and kinesin-1C are neuron specific, whereas kinesin-1B ("conventional kinesin") is expressed ubiquitously [5-7]. Little is known about the cargoes of kinesin-1A, though potential cargoes and interactors include HAP-1 (huntingtin associated protein-1) [8], DISC-1 (disrupted in schizophrenia protein-1) and the NUDEL/LIS1/14-3-3ε complex [9], Grb2 (growth factor receptor bound protein-2) [10], and β-dystrobrevin [11,12].

Neurofilaments are the intermediate filaments of neurons. They are heteropolymers of variable composition and subunit stoichiometry, typically composed of the low, medium and high molecular weight neurofilament triplet proteins (NFL, M and H), as well as peripherin and/or alpha-internexin [13]. Live-cell imaging of neurofilament transport in cultured neurons and computational modeling studies of neurofilament transport *in vivo *have demonstrated that neurofilaments move along axons in a rapid, intermittent and bidirectional manner [14-17].

Studies on kinesin-1A knockout mice have suggested that kinesin-1A may be an anterograde motor for neurofilaments in axons [18]. In support of this proposal, we observed a 75% reduction in the frequency of neurofilament movement in cultured neurons from kinesin-1A knockout mice [19]. Interestingly, both anterograde and retrograde movement was affected and movement in both directions could be rescued by kinesin-1A. Partial rescue was also observed with kinesin-1B and kinesin-1C, though with successively decreasing efficacy. In addition, headless kinesin-1A and kinesin-1C each inhibited both anterograde and retrograde neurofilament transport in a dominant-negative manner in wild type neurons. Because dynein is thought to be the retrograde motor for axonal neurofilaments, we investigated the effect of dynein inhibition on neurofilament transport. Disruption of dynein function by using RNA interference, dominant- negative approaches, or a function-blocking antibody also inhibited both anterograde and retrograde neurofilament movement. These data suggest that kinesin-1A is the principal but not exclusive anterograde motor for neurofilaments, that there may be some functional redundancy among the kinesin-1 isoforms with respect to neurofilament transport, and that the activities of the anterograde and retrograde neurofilament motors are tightly coupled.

**Figure 1:**
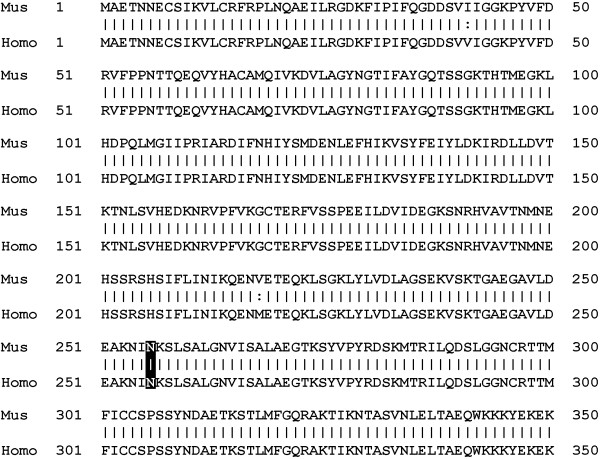
**Sequence comparison of mouse and human kinesin-1A**. Sequence alignment of kinesin-1A in mice (Genbank Accession number NP_001034089) and humans (Genbank Accession number NP_004975). The motor domain spans residues 1 through 336 and is identical except for conserved substitutions at residues 41 and 218. The N256S mutation occurs at an invariant asparagine residue (highlighted in inverted contrast) located in the switch II loop/helix motif of the microtubule binding site.

Since kinesin-1A appears to be a motor for neurofilaments, we have investigated the effect of an SPG10 mutation in this motor on neurofilament transport in cultured neurons. Of the 16 different SPG10 mutations that have been identified to date, 15 reside in the kinesin motor domain and 14 of these are missense mutations [20-26]. A particular hot spot for these mutations is in the vicinity of the microtubule and nucleotide binding sites. In the present study, we have focused on the N256S mutation, which results in the substitution of a highly conserved asparagine residue in the switch II loop/helix motif of the microtubule binding site [20] (Figure 1). We show that expression of N256S-kinesin-1A disrupts both anterograde and retrograde neurofilament neurofilament transport in cultured mouse cortical neurons, raising the possibility that neurofilament transport may also be disrupted in patients with SPG10.

## Results

**Figure 2:**
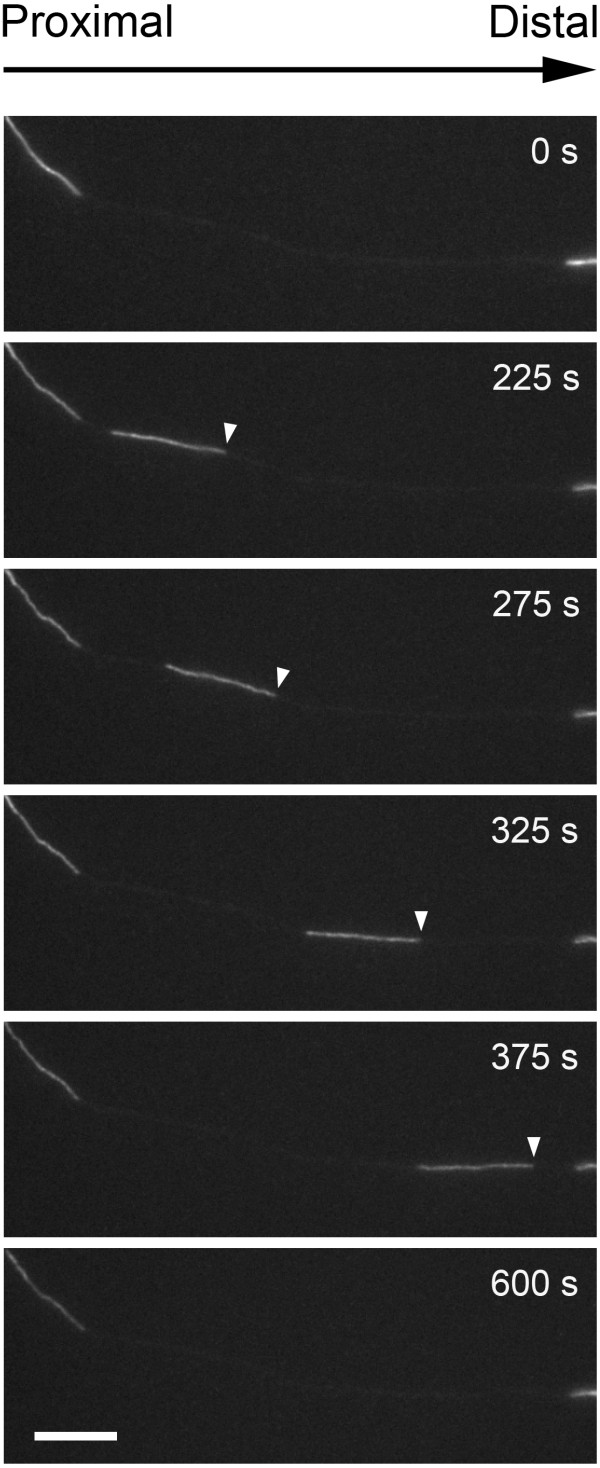
**A neurofilament moving through a gap in the axonal neurofilament array**. Axon of a cortical neuron expressing GFP-NFM, visualized by epifluorescence microscopy. The axons exhibit discontinuities in the axonal neurofilament array, which we call gaps. Filaments that move into the gaps can be tracked by time-lapse imaging to analyze the kinetics of movement. These images are excerpted from the movie named Additional file 3 in the Supplementary Data. Proximal is left and distal is right. Scale bar = 10 μm.

To study the effect of N256S-kinesin-1A on neurofilament transport, we transfected cultured mouse cortical neurons with GFP-tagged neurofilament protein M (GFP-NFM) with or without mutant or wild type mouse kinesin-1A. The purpose of the wild type kinesin-1A was to control for possible effects due to overexpression of the motor. Mouse cortical neurons exhibit gaps in the axonal neurofilament array similar to those that we have observed in neurons from superior cervical ganglia [14-19], but the gaps in the cortical neurons are longer and more numerous, making these cells particularly suitable for studies of neurofilament movement (Figure 2). The GFP-NFM fusion protein incorporates throughout all the neurofilaments in these cells, permitting all neurofilaments to be detected. Additional files 1 &2 in the Supplementary Data are examples of movies showing the abundant neurofilament movement that can be observed in these neurons.

**Figure 3:**
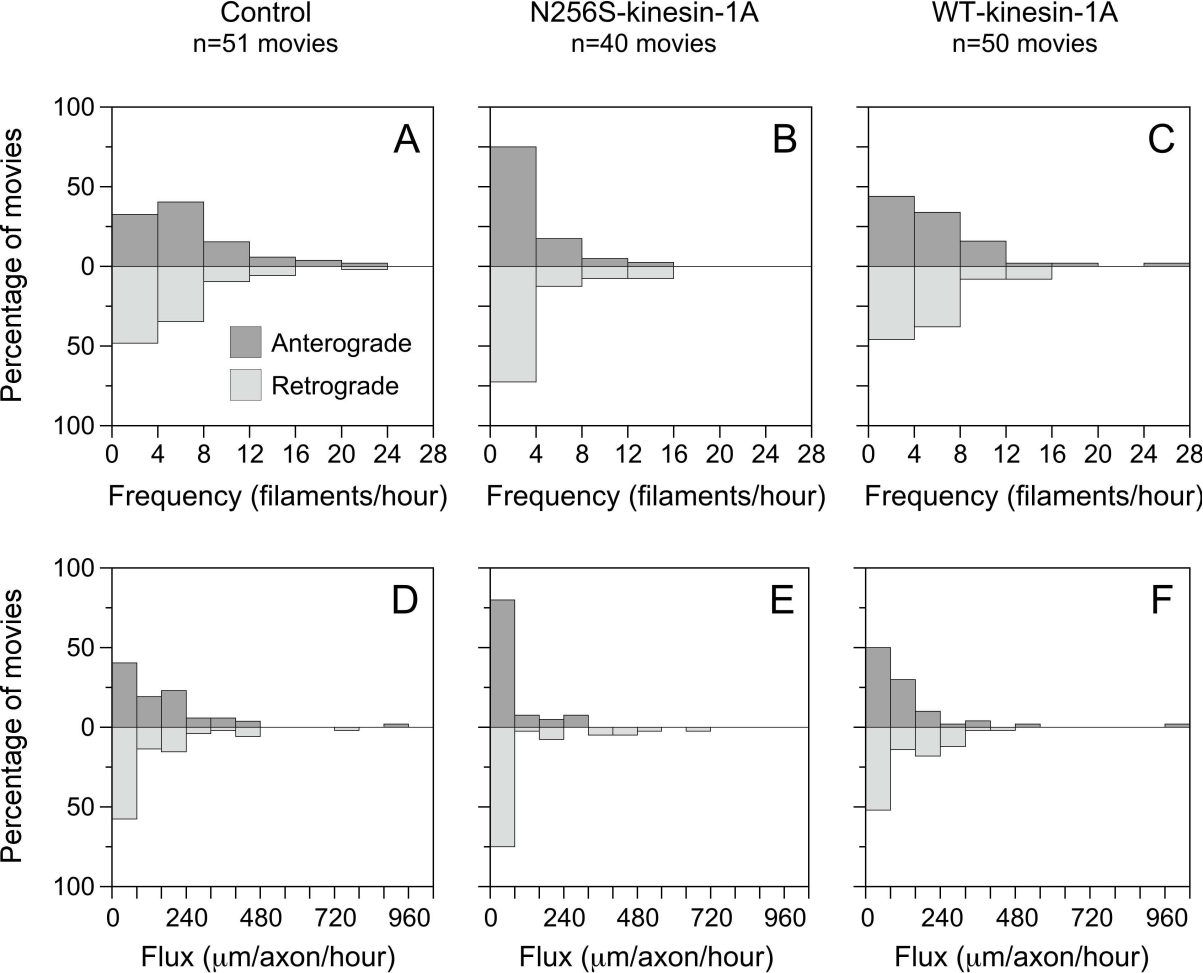
**Effect of N256S-kinesin-1A on the frequency and flux of neurofilament movement**. Analysis of neurofilament movement through gaps in axons of cortical neurons expressing GFP-NFM alone (A & D; 51 movies), GFP-NFM plus N256S-kinesin-1A (B & E; 40 movies), or GFP-NFM plus exogenous wild type kinesin-1A (C & F; 50 movies). (A-C) Histograms of the frequencies of neurofilament movement, expressed as number of neurofilaments per hour. The filaments were classified as anterograde or retrograde based on their preferred direction of movement and an anterograde and retrograde frequency was calculated for each movie. (see Methods). (D-F) Histograms of the neurofilament fluxes, expressed as total distance moved by all the filaments per axon per hour in either the anterograde or retrograde direction. An anterograde and retrograde flux was calculated for each movie.

To track the movement of the GFP-tagged neurofilaments, we observed gaps by epifluorescence microscopy and acquired time-lapse movies using one second exposures at four second time intervals. Each movie was exactly 15 minutes in length. Ninety six percent of the moving structures were filamentous in shape, ranging from 1.3 μm to 44.5 μm in length and diffraction limited in width. The average length was 8.6 μm, which is comparable to what we observed in previous studies on neurons from mouse superior cervical ganglia [19-27]. We defined transport frequency as the number of filaments that moved at least 50 pixels (6.55 μm) per 15-minute movie. N256S-kinesin-1A reduced the average neurofilament transport frequency significantly, from 4.5 to 1.4 filaments/hour in the anterograde direction (p < 0.001) and from 3.2 to 2.0 filaments/hour in the retrograde direction (p = 0.047; Figure 3). Expression of wild type kinesin-1A reduced the average neurofilament transport frequency from 4.5 to 3.7 filaments/hour anterogradely and from 3.2 to 3.1 filaments/hour retrogradely, but these effects were not statistically significant (p = 0.26 and p = 0.92, respectively). Thus N256S-kinesin-1A impaired neurofilament transport in both anterograde and retrograde directions in these axons. Additional files 3, 4 &5 in the Supplementary Data are examples of the movies obtained in these experiments.

To analyze the motility defect in more detail, we tracked the movement of each filament through successive frames of the time-lapse movies. We defined anterograde and retrograde transport flux as the total distance moved in the corresponding direction by all the filaments in each 15-minute movie. Since each movie contained a single axon, we expressed the fluxes in units of μm/axon/hour. N256S-kinesin-1A reduced the average neurofilament transport flux from 149 to 46 μm/axon/hour in the anterograde direction (p < 0.001) and from 116 to 90 μm/axon/hour in the retrograde direction (p = 0.007; Figure 3). Expression of wild type kinesin-1A reduced the transport flux from 149 to 109 μm/axon/hour anterogradely and from 116 to 105 μm/axon/hour retrogradely, but these reductions were not statistically significant (p = 0.13 and p = 0.69, respectively). Thus, in addition to decreasing the frequency of neurofilament movement, N256S-kinesin-1A also decreased the total extent of neurofilament movement.

**Figure 4:**
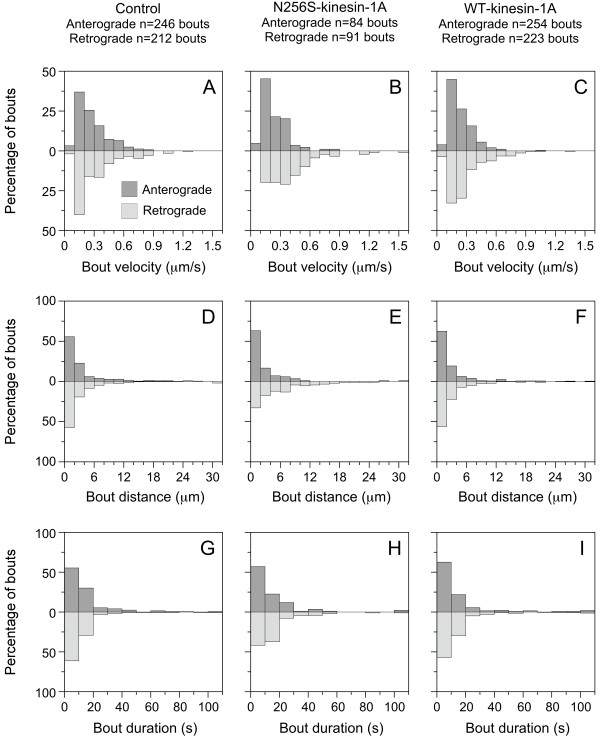
**Effect of N256S-kinesin-1A on the velocity, distance and duration of bouts of neurofilament movement**. Analysis of the velocity, distance and duration of bouts of neurofilament movement in axons of cortical neurons expressing GFP-NFM alone (A, D & G; 246 anterograde bouts, 212 retrograde bouts), GFP-NFM plus N256S-kinesin-1A (B, E & H; 84 anterograde bouts, 91 retrograde bouts), or GFP-NFM plus exogenous wild type kinesin-1A (C, F & I; 254 anterograde bouts, 223 retrograde bouts). The anterograde and retrograde bouts are represented by the dark grey and light grey bars, respectively. The y-axis represents the percentage of bouts in the corresponding direction. Note: to avoid compressing the scale on the x-axis, the right-most bin represents an expanded bin size of 30-50 in graphs D-F, and 100-500 in graphs G-I.

**Figure 5:**
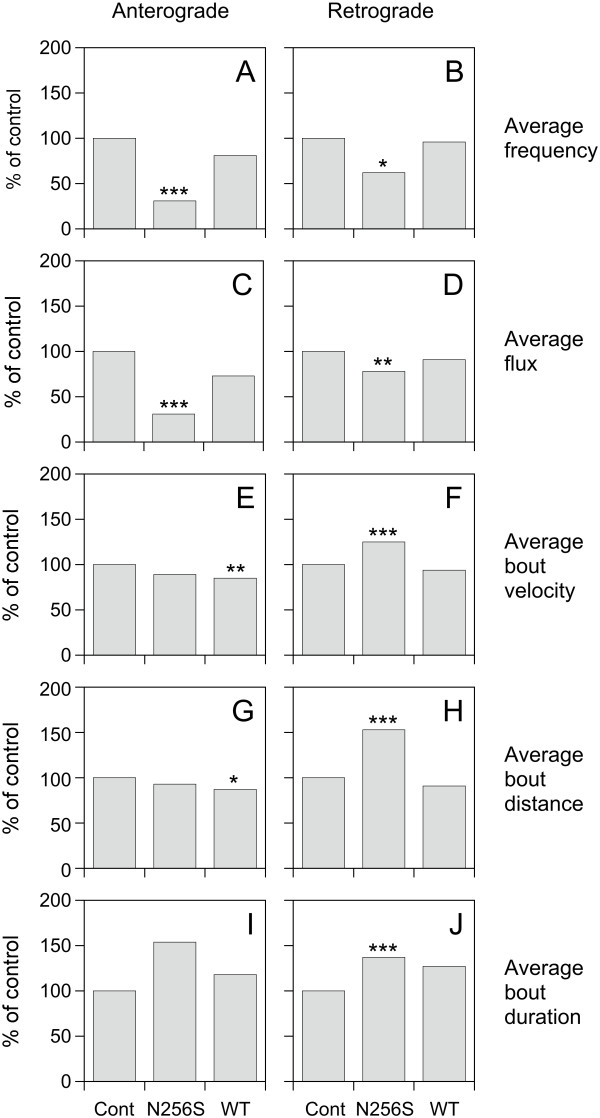
**Summary of kinetic data**. Bar graphs summarizing the kinetics of neurofilament movement in axons of cortical neurons expressing GFP-NFM alone, GFP-NFM plus N256S-kinesin-1A, or GFP-NFM plus exogenous wild type kinesin-1A. Graphs A-D summarize the data in Figure 3 and graphs E-J summarize the data in Figure 4. The asterisks denote the p values comparing the GFP-NFM plus N256S-kinesin-1A and the GFP-NFM plus exogenous wild type kinesin-1A to GFP-NFM alone (Mann-Whitney test: *** = p < 0.005; ** = p < 0.01; * = p < 0.05).

To determine whether N256S-kinesin-1A also affected the velocity or persistence of neurofilament movement, we measured the velocity, distance and duration of each bout of movement for each moving filament. We defined a bout as a period of uninterrupted movement between two pauses or between a pause and a reversal. N256S-kinesin-1A decreased the average bout velocity from 0.27 μm/s to 0.24 μm/s in the anterograde direction, but this was not statistically significant (p = 0.053; Figure 4). In contrast, N256S-kinesin-1A increased the average retrograde bout velocity from 0.32 to 0.40 μm/s, and this was statistically significant (p < 0.001). N256S-kinesin-1A also increased the average retrograde bout distance from 3.7 μm to 5.7 μm (p < 0.001) and the average retrograde bout duration from 11 seconds to 15 seconds (p < 0.001), but without any significant effect on average anterograde bout distance (3.2 μm, p = 0.36) or average anterograde bout duration (19 seconds, p = 0.98). Expression of wild type kinesin-1A had no significant effect on average retrograde bout velocity (0.30 μm/s, p = 0.76), average retrograde bout distance (3.4 μm, p = 0.57), or average retrograde bout duration (14 seconds, p = 0.32). Expression of wild type kinesin-1A did decrease the average anterograde bout velocity from 0.27 μm/s to 0.23 μm/s (p = 0.006) and average anterograde bout distance from 3.4 μm to 3.0 μm (p = 0.043), but without any significant effect on average anterograde bout duration (15 seconds, p = 0.31). Thus, N256S-kinesin-1A reduced the anterograde flux by decreasing anterograde frequency without affecting anterograde velocity. N256S-kinesin-1A also reduced the retrograde flux in spite of an increase in retrograde velocity because the increase in retrograde velocity was not sufficient to compensate for the decrease in retrograde frequency (summarized in Figure 5).

**Figure 6:**
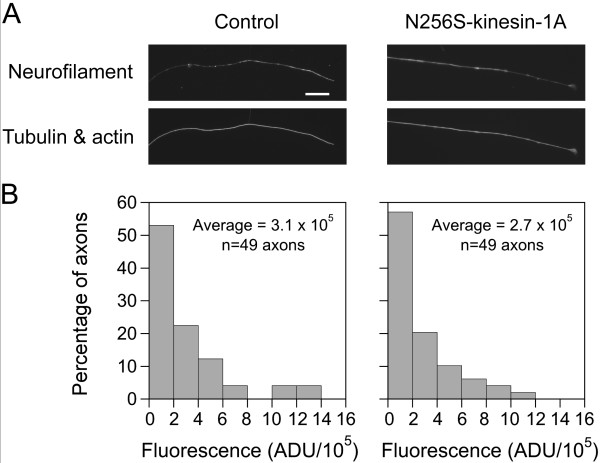
**N256S-kinesin-1A does not deplete neurofilaments from distal axons**. Comparison of the neurofilament content of distal axons from cortical neurons expressing GFP-NFM alone (control) or GFP-NFM plus N256S-kinesin-1A. (A) Immunofluorescence microscopy for neurofilament protein (top panels) and for tubulin and actin (bottom panels; see Methods). Scale bar = 20 μm. (B) Quantification of neurofilament content in distal axons. There was no significant difference in the neurofilament content of axons expressing GFP-NFM plus N256S-kinesin-1A (49 axons) compared to control axons expressing GFP-NFM alone (49 axons; p = 0.73, Mann-Whitney test).

We have shown previously that neurofilaments are delivered to distal axons by anterograde movement and retrieved by retrograde movement [27]. Thus it is possible that the decrease in retrograde flux described above could be explained by a depletion of neurofilaments from distal axons as a secondary consequence of the disruption of anterograde movement. To test this hypothesis, we transfected cultured cortical neurons with GFP-NFM, either with or without N256S-kinesin-1A, and then fixed and processed the cells for immunofluorescence microscopy after 10-11 days in culture using antibodies specific for GFP and neurofilament protein M (NFM). Since axonal neurofilament distribution is discontinuous in these neurons, we also stained for tubulin and actin, which are present along the entire length of each axon. Cells expressing N256S-kinesin-1A were identified by their GFP expression. To quantify neurofilament content, we measured the fluorescence intensity of NFM in distal axons, extending 100 μm proximally from the base of the growth cone. There was no statistically significant difference in the neurofilament content of axons expressing N256S-kinesin-1A compared to axons expressing no exogenous kinesin-1A (Figure 6). Thus, disruption of neurofilament transport by N256S-kinesin-1A does not appear to deplete distal axons of neurofilaments, and therefore the reduction in retrograde neurofilament flux cannot be a secondary consequence of the disruption of anterograde neurofilament movement. The absence of a reduction in neurofilament content in distal axons expressing N256S-kinesin-1A is probably due to the impairment of both anterograde and retrograde neurofilament movement, which would be expected to impair both delivery and departure of neurofilaments from these axonal regions.

**Figure 7:**
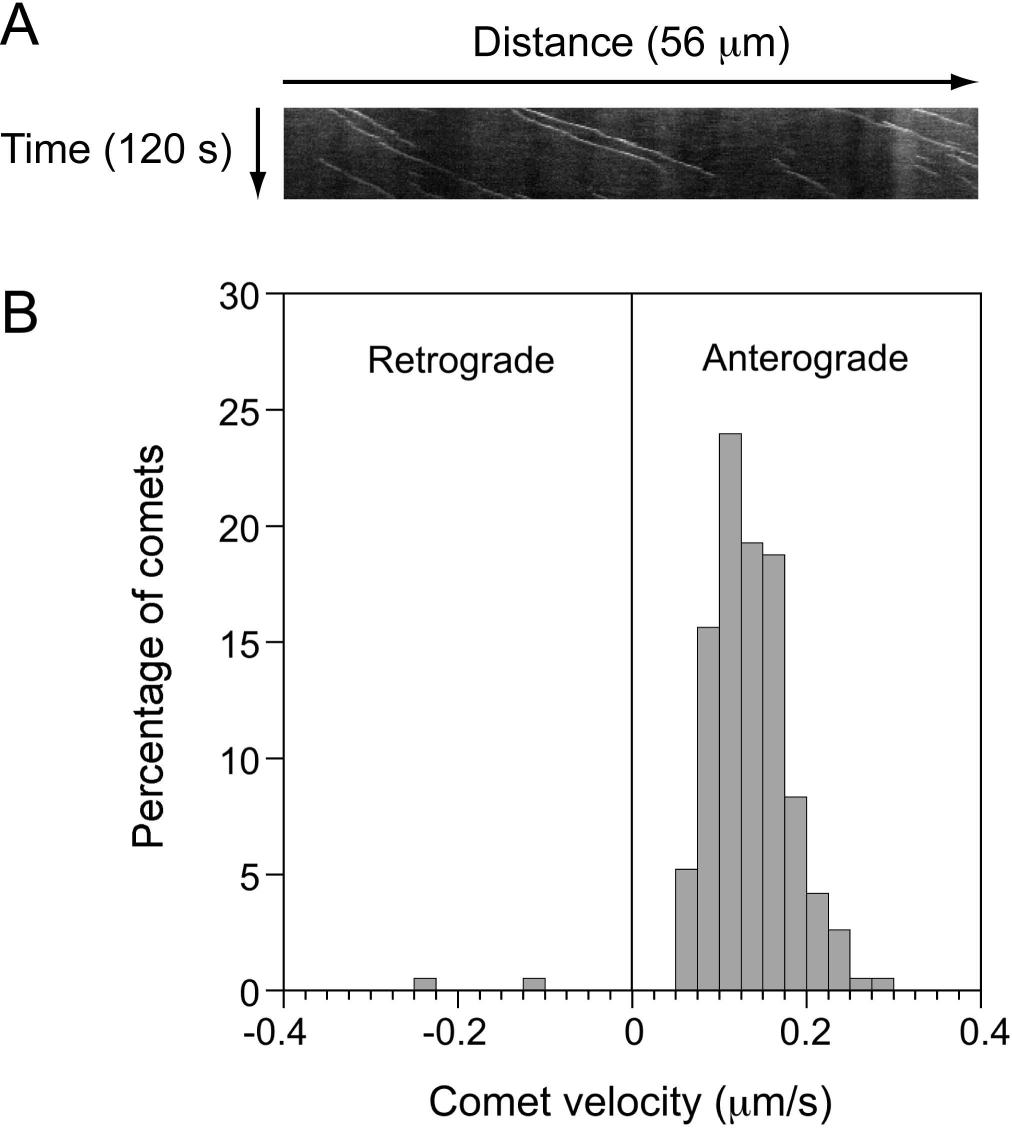
**Microtubules in cortical neuron axons are plus-end distal**. Cortical neurons were transfected with YFP-EB1, which is a protein that binds to the growing plus ends of microtubules. Axons were imaged for 2 minutes at 2 second intervals in order to determine the direction of microtubule growth. (A) A typical kymograph showing a number of YFP-EB1 comets moving anterogradely. The horizontal dimension represents distance and the vertical dimension represents time. This kymograph was generated from the movie named Additional file 6 in the Supplementary Data. (B) Histogram of YFP-EB1 comet velocities (192 comets from 23 different neurons). Note that almost all the comets move anterogradely, confirming the plus-end distal orientation of the microtubules in these axons.

Microtubules in axons are widely accepted to be orientated exclusively with their plus-ends distal, and kinesin-1 motors are known to move cargoes exclusively toward the plus-ends of these polymers. Thus the disruption of retrograde neurofilament movement by N256S-kinesin-1A suggests that this mutant also disrupts minus-end directed neurofilament movement, which is thought to be mediated by dynein [28-31]. However, another possibility, albeit unlikely, is that microtubule polarity in cultured mouse cortical axons is not entirely or predominantly plus-end distal. To test this hypothesis, we transfected cultured cortical neurons with the microtubule plus-end tracking protein EB1 tagged with yellow fluorescent protein (YFP-EB1) and imaged the movement of YFP-EB1 comets. Additional file 6 in the Supplementary Data is an example of a movie showing YFP-EB1 comet movement in these cells. Using kymograph analysis, we tracked 192 comets in 23 axons. 190 comets moved anterogradely and 2 comets moved retrogradely (Figure 7). The average comet velocity was 0.13 μm/s, which is consistent with published estimates of the rate of microtubule growth in cells [32]. These data confirm that microtubules in these axons are indeed almost exclusively plus-end distal (especially considering that the plus-end proximal microtubules could represent microtubules that looped back on themselves). Thus the disruption of retrograde neurofilament transport by N256S-kinesin-1A appears to be due to disruption of minus-end directed movement by this mutant plus-end directed motor.

## Discussion

The SPG10 form of hereditary spastic paraplegia is an autosomal dominant disease caused by mutations in the kinesin-1A motor protein. Since there is evidence that kinesin-1A is a motor for neurofilaments, we investigated the effect of an SPG10 mutant, N256S-kinesin-1A, on neurofilament transport in cultured neurons. Expression of N256S-kinesin-1A in cultured mouse cortical neurons impaired neurofilament transport in both anterograde and retrograde directions, but unexpectedly this was due primarily to a decrease in the frequency, not the velocity of movement. A limitation of our experimental approach is that transient transfection of the mutant motor does not permit quantification or regulation of the expression level relative to the endogenous wild type motor. Therefore, to control for possible effects of over-expression, we also characterized neurofilament movement in neurons transfected with wild type kinesin-1A motor. Simply over-expressing wild type kinesin-1A had a small effect on anterograde bout velocity and anterograde bout distance, but no effect on average frequency or flux in either the anterograde or retrograde direction. Thus the effects of the N256S-kinesin-1A on neurofilament transport were due to the N256S mutation and were not an artifact of over-expression.

The impairment of both anterograde and retrograde neurofilament movement in these experiments is notable because kinesin-1A is an anterograde motor in axons, but this result is consistent with recent evidence from our laboratory showing that the anterograde and retrograde neurofilament motors are interdependent [19]. In that study, we found that both anterograde and retrograde neurofilament movement were impaired in neurons from kinesin-1A knockout mice, and that expression of wild type kinesin-1A rescued the movement in both directions. In addition, expression of a headless dominant negative kinesin-1A construct in wild type neurons impaired both anterograde and retrograde neurofilament movement, and disruption of dynein function by using RNA interference, dominant negative approaches, or a function-blocking antibody also inhibited both anterograde and retrograde neurofilament movement. Thus there is functional coupling between kinesin-1A and dynein motors in the bidirectional transport of neurofilaments along microtubules in axons.

The mechanism by which microtubule motors of opposing directionality interact to regulate bidirectional cargo transport is not yet understood. Two favored models are the tug-of-war model and the coordination model [33]. In the tug-of-war model, the direction of movement is the result of a dynamic competition between opposing motors that are bound and active at the same time. In the coordination model, the opposing motors interact so that only motors of one directionality are bound or active at one time. In their simplest form, these two models have quite different predictions: in the tug-of-war model, impairment of motors of one directionality should increase the velocity and frequency of movement in the opposite direction, whereas in the coordination model it should not. In fact, according to the coordination model, manipulations or mutations that disrupt the coordination could also cause the motors to interfere with each other, leading to impairment of movement in both directions [34]. Many labs have reported reciprocal inhibition of both directions of movement after disruption of motors of one directionality, which is consistent with the coordination model (see [19] for citations to these studies).

In the present study, we found that expression of N256S-kinesin-1A reduced the frequency and flux of neurofilament movement in both anterograde and retrograde directions and increased bout velocity, distance and duration in the retrograde direction. It is unclear how to interpret these data mechanistically. The decrease in both anterograde and retrograde frequency and flux suggests a coordination mechanism, but the increase in retrograde bout velocity, distance and duration suggests a tug-of-war. Thus it is likely that the mechanism is more complex, perhaps combining features of both models. For example, Lipowsky and colleagues have shown that tug-of-war models that account for the load-dependence of the interaction between motors and their tracks can generate bouts of persistent anterograde and retrograde movement, depending on the relative numbers of bound motors [35,36]. To test such hypotheses in the case of neurofilaments it will be necessary to record neurofilament movement with much higher spatial and temporal resolution than we have done in the present study, and also to measure the forces acting on the moving filaments, which is currently not possible because neurofilaments are too small to be optically trapped.

Studies *in vitro *suggest that the N256S-kinesin-1A mutant is a defective motor and that it may act as a dominant-negative disruptor of kinesin-1A transport. Mutation of the homologous amino acid residue to a lysine in the motor domain of the yeast kinesin-14 motor kar3 (N650K-kar3) or in the motor domain of the fungal kinesin-14 motor ncd (N600K-ncd), prevents microtubule-stimulated activation of the motor ATPase by uncoupling nucleotide and microtubule binding [37]. Both N650K-kar3 and N600K-ncd are capable of binding and hydrolyzing ATP, but in contrast to wild type kinesin, they bind tightly to microtubules in both the ATP-bound and ADP-bound states. In microtubule gliding assays *in vitro*, N600K-ncd motors bind microtubules but do not translocate them. In yeast cells, N650K-kar3 exhibits a dominant negative effect over wild-type kar3 [38]. *In vitro*, N256S-kinesin-1A is unable to generate single-motor processive motion in microtubule gliding and bead motility assays, resulting in decreased gliding and transport velocities [39]. However, in contrast to the N to K mutations in N650K-kar3 and N600K-ncd, the N256S mutation in kinesin-1A did not bind microtubules in rigor. When N256S-kinesin-1A was mixed with wild type kinesin-1A in microtubule gliding and particle motility assays *in vitro*, the mutant kinesin appeared to exert a dominant inhibitory effect on the transport velocity. Given these observations, however, it is surprising that the disruption of neurofilament transport by N256S-kinesin-1A in our study was due primarily to a decrease in the frequency. There was a slight decrease in average velocity (from 0.27 to 0.24 μm/s), but this was not statistically significant. Moreover, this effect was probably an artifact of over-expression, since we observed a similar decrease in cells expressing wild type kinesin-1A. Thus the behavior of the motor in motility assays *in vitro *cannot necessarily predict its effect on cargo transport *in vivo*.

Neurofilaments accumulate abnormally and excessively in many neurodegenerative diseases, including amyotrophic lateral sclerosis, giant axonal neuropathy, and Charcot Marie Tooth disease [40,41]. Several studies have suggested that these accumulations may arise due to perturbations in axonal transport [42-44]. In support of this idea, slowing of axonal transport has been reported in mouse models of SOD-mediated amyotrophic lateral sclerosis [45] and is an early event in the progression of this disease [46]. Disruption of axonal transport by over-expression of dynamitin in neurons also results in accumulations of neurofilaments [47], and mutations in dynein subunits are one cause of motor neuron disease in humans [48,49]. Defects in axonal transport have also been reported in mouse models of spastin-mediated hereditary spastic paraplegia (SPG4) [50,51], and swellings containing vesicular and cytoskeletal proteins, including neurofilament proteins, have been reported in humans with this disease [50,51].

While it is clear that the axonal transport of neurofilaments is impaired in many neurodegenerative diseases, the role of neurofilament accumulations in the disease progression has been controversial. Over-expression of the human high molecular weight neurofilament protein (NFH) in mice causes a slowing of neurofilament transport, accumulations of axonal neurofilaments, and motor neuron degeneration [42-52]. However, the significance of these studies is unclear because over-expression of mouse NFH has no pathological effects [53]. One approach to test the role of neurofilaments in mouse models of neurodegenerative diseases has been to cross the mice with neurofilament L knockout mice, which lack neurofilament polymers. Using this approach, it has been shown that the absence of neurofilaments in neurons slows the progression of disease dramatically in mouse models of superoxide dismutase (SOD)-mediated ALS [54,55]. On the other hand, similar experiments using a transgenic mouse expressing an NFH β-galactosidase fusion protein, which aggregates and sequesters neurofilaments in neuronal cell bodies, showed no significant alteration of disease pathology in mouse models of dystonia musculorum and SOD-mediated ALS, though there was some prolongation of neuronal survival and some delay of axon loss [56].

Apparently contradictory results on the role of neurofilaments in disease have also been obtained in experiments on the accumulation of neurofilaments in response to neurotoxins such as acrylamide and hexanedione. These agents impair or accelerate axonal transport of neurofilaments and other cargoes and lead to focal accumulations and depletions of axonal neurofilaments. Studies with transgenic mice expressing NFH β-galactosidase fusion protein suggest that neurofilaments are not essential for the toxicity associated with the administration of these substances [57], whereas studies on the Quiverer (Quv) quail, which lack NFL, suggest that they are [58]. Perhaps the conflicting nature of these reports may be due to differences between the animal models used. For example, the NFH β-galactosidase transgenic mice have perikaryal neurofilament accumulations whereas the NFL knockout mice and the Quiverer quail do not. Whatever the explanation, however, it does seem clear that the accumulation of neurofilaments can be an exacerbating factor in at least some circumstances.

Neurofilaments are unlikely to be the sole cargo for kinesin-1A in neurons so it is possible that deficiencies in the movement of other cargoes may contribute to the disease progression in SPG10. Moreover, while neurofilament accumulations have been described in patients with SPG4 (see above), there have been no ultrastructural studies on nerves of patients with SPG10. Thus it is presently unclear whether neurofilament accumulations are a feature of this disease. In the present study we did not observe local neurofilament accumulations in axons of neurons expressing N256S-kinesin-1A. However, it is unclear to what extent such observations in short term cultures can predict the long-term effects of SPG10 mutaions on neurofilament organization *in vivo*. For example, it is quite possible that subtle changes in neurofilament organization or distribution in short-term cultures of neonatal cultured neurons might be magnified over longer time scales in mature neurons *in vivo*. Either way, the fact that kinesin-1A appears to be a neurofilament motor and that N256S-kinesin-1A disrupts the bidirectional transport of neurofilaments in cultured neurons suggest that patients with the SPG10 form of hereditary spastic paraplegia may well have neurofilament transport abnormalities which may contribute to the disease progression, and this warrants further investigation.

## Conclusions

Mutations in kinesin-1A, which is a putative anterograde motor for axonal neurofilaments, cause the SPG10 form of hereditary spastic paraplegia. We investigated the effect of an SPG10 point mutation in kinesin-1A on neurofilament transport in cultured mouse cortical neurons. We showed that this mutant disrupts both anterograde and retrograde neurofilament transport, raising the possibility that neurofilament transport may also be disrupted in patients with SPG10.

## Materials and methods

### Molecular Cloning

Mouse kinesin-1A cDNA (Genbank accession No. BC058396, I.M.A.G.E. clone 6824963) was obtained from American Type Culture Collection (Manassas, VA) and then subcloned into pEGFP-C1 (Clontech, Mountain View, CA) lacking the EGFP sequence, as previously described [19]. The N256S-kinesin-1A plasmid construct was generated using a QuikChange Site-Directed Mutagenesis Kit (Stratagene, La Jolla, CA) with forward primer 5'-GGC AAA GAA TAT CAG CAA GTC GCT GTC GGC CC and reverse primer 5'-GGG CCG ACA GCG ACT TGC TGA TAT TCT TTG CC. The wild type and mutant kinesin-1A constructs were tagged with cMyc at their N-terminus with forward primer 5'-CTA GCT CCG GAA TGG AGC AGA AGC TGA TCA GCG AGG AGG ACC TGG AG and reverse primer 5'-TCG ACT CCA GGT CCT CCT CGC TGA TCA GCT TCT GCT CCA TTC CGG AG. The resulting cMyc-N256S-kinesin-1A and cMyc-kinesin-1A constructs were confirmed by DNA sequencing of their open reading frames. The EGFP-mNFM plasmid construct, which encodes the codon-optimized F64L/S65T variant of green fluorescent protein attached to the amino terminus of mouse neurofilament protein M, was described by Yan et al. [59]. The YFP-EB1 plasmid construct was provided by Chen Gu [60].

### Cell culture

Cortical neurons were cultured using the glial sandwich technique of Banker [61]. To prepare glial cultures, the cerebral cortices of 3 to 5 P0 mice were dissociated in phosphate buffered saline (PBS; Invitrogen, Carlsbad, CA) containing 0.25% [w/v] trypsin (Worthington Biochemical Corp., Lakewood, NJ), 1% [w/v] DNase-I (Sigma, St. Louis, MO) and 0.54mM EDTA (Sigma) and the cells were cultured in plastic dishes at 37°C/5% CO_2 _in glial medium, which consisted of Minimum Essential Medium (Invitrogen) supplemented with 10% [v/v] horse serum (Invitrogen), 0.7% [w/v] glucose (Sigma) and 16 μg/ml gentamicin (Invitrogen). The cells were typically passaged 5-10 times and then cryopreserved for future use. To prepare neuronal cultures, the cerebral cortex of one P0 mouse was dissociated in PBS containing 0.025% [w/v] Trypsin, 0.27mM EDTA (Sigma) and 0.5% [w/v] DNase-I. The dissociated cells were plated onto glass-bottomed dishes that had been coated with poly-D-lysine (Sigma) and laminin (BD Biosciences, San Jose, CA). Glass coverslips bearing glia (>80% confluency) were suspended over the neurons using dots of paraffin wax as spacers, and the resulting sandwich cultures were maintained initially at 37°C/5% CO_2 _in plating medium, which consisted of Neurobasal medium (Invitrogen) supplemented with 2% [v/v] B-27 Supplement Mixture (Invitrogen), 0.27% [w/v] glucose, 2 mM glutamine (Invitrogen), 37.5 mM NaCl (Sigma), 5% [v/v] fetal bovine serum (FBS; Thermo Scientific, Waltham, MA), 16 μg/ml gentamicin, and 2.5 μM cytosine arabinoside (AraC; Sigma). After two days, the plating medium was replaced with culturing medium, which was identical to the plating medium except that it lacked serum. Every four days, half the medium was removed and replaced with fresh medium.

### Transfection

The dissociated cortical neurons were transfected by electroporation prior to plating using an Amaxa Nucleofector™ (Lonza Inc., Walkersville, MD) with the mouse neuron nucleofection kit (VPG-1001) and program O-05. The volume of the cell suspension was 100 μl and the cell density ranged from 4 × 10^6 ^to 6 × 10^6 ^cells/ml. For the experiments on neurofilament movement and distribution, we used 2 μg EGFP-mNFM construct either alone or in addition to 2 μg N256S-kinesin-1A or 2 μg wild type kinesin-1A construct. For the microtubule polarity experiments, we used 2 μg YFP-EB1 construct.

### Live-cell imaging

To image neurofilament movement, cortical neurons were observed after 8 to 12 days in culture by epifluorescence microscopy on a Nikon TE300 inverted microscope (Nikon, Garden City, NY) using a 100x Plan Apo VC 1.4NA oil immersion objective. The observation medium consisted of Hibernate-E (BrainBits, Springfield, IL) supplemented with 2% [v/v] B27 Supplement Mixture, 0.3% [w/v] glucose, 1 mM L-glutamine, 37.5 mM NaCl, and 10 μg/ml gentamicin. The temperature on the microscope stage was maintained using an Air Stream incubator (Nevtek, Williamsville, VA). A layer of dimethylpolysiloxane fluid (Sigma, 5 centistokes) was floated over the observation medium to prevent evaporation. For time-lapse imaging, the exciting light from the mercury arc lamp was attenuated 12-fold using neutral density filters, and images were acquired with one second exposures at four second intervals using a Micromax 512BFT cooled CCD camera (Roper Scientific, Trenton, NJ) and MetaMorph™ software (Molecular Devices, Sunnyvale, CA). All movies were 15 minutes in length. It was necessary to adjust the focus occasionally during movie acquisition to correct for focus drift. To image YFP-EB1 comets, cortical neurons were observed after 8-10 days in culture by epifluorescence microscopy using a Nikon TE2000 microscope. For these experiments, the exciting light from the mercury arc lamp was attenuated 4-fold using neutral density filters, and images were acquired with one second exposures at two second intervals for two minutes using a CoolSNAP HQ cooled CCD camera and 2 × 2 pixel binning (Roper Scientific). For publication, the movies were saved in QuickTime format using the H.264 video codec.

### Motion analysis

Neurofilament movement was analyzed by tracking the position of the leading or trailing ends of the filaments in successive frames of the time-lapse image movies using MetaMorph™ software. All objects greater than or equal to 10 pixels (1.31 μm) in length were analyzed if they moved a total distance of at least 50 pixels (6.55 μm) and could be tracked through at least three successive frames of the movie. To calculate the frequency of movement, we classified each neurofilament as anterograde or retrograde based on its preferred direction of motion and then counted the number of anterograde and retrograde moving filaments per movie. Thus for each movie we obtained two frequency measurements, one anterograde and one retrograde. Ninety seven percent of the filaments exhibited a preferred direction of movement, defined as moving at least 70% of their time in the same direction. The remaining 3% of the filaments, which spent more than 30% of the time moving in the opposite direction, were each considered to represent separate anterograde and retrograde moving events. To calculate the flux, we grouped all the filaments in each 15-minute movie together and measured the total anterograde and retrograde distance moved. Thus for each movie we obtained two flux measurements, one anterograde and one retrograde. For the analysis of bout velocity, bout duration and bout distance, we defined a bout of movement to be a phase of uninterrupted movement between two pauses or between a pause and a reversal. Thus each bout velocity represents the bout distance divided by the bout duration. Bouts in which the filament was moving at the start or end of the movie were ignored because their true duration could not be assessed. Statistical comparisons were performed using the Mann-Whitney test.

### Immunofluorescence microscopy

Cells were fixed with 4% paraformaldehyde ten or eleven days after plating and then extracted with PBS containing 1% [v/v] Triton X-100 and 0.3 M NaCl. For immunostaining, the cells were stained first with a rabbit polyclonal specific for NFM (AB1987; Chemicon, MA, 1:200) and then subsequently with a mouse monoclonal antibody specific for beta-tubulin (N357; Amersham, NJ, 1:400) and a goat polyclonal antibody specific for GFP (Goat anti-GFP; Rockland, 1:2000). The secondary antibodies were Alexa 488-donkey anti-goat (Invitrogen, 1:200), Alexa 568-donkey anti-mouse (Invitrogen, 1:200), and Alexa 647-donkey anti-rabbit (Invitrogen, 1:200). Actin was visualized by including Alexa-568 phalloidin (Invitrogen, 1:20) in the secondary antibody mixture. Coverslips were mounted using ProLong Gold Antifade reagent (Invitrogen). Images were acquired on a Nikon TE2000 microscope with a 40x Plan Apo 1.0 NA oil immersion objective and a CoolSNAP HQ cooled CCD camera. The epifluorescent illumination was attenuated 4-fold using neutral density filters, and images were acquired with 100 millisecond exposures. To quantify the fluorescence in the distal axon, we measured the fluorescence intensity in the most distal 100 μm of each axon, extending proximally from the base of the growth cone.

## Competing interests

The authors declare that they have no competing interests.

## Authors' contributions

LW performed the experiments, analyzed the data, and prepared the figures. AB conceived of the study and worked closely with LW to design the experiments and to analyze and interpret the data. Both authors participated in the writing, and both authors read and approved the final manuscript.

## Supplementary Material

Additional file 1 **Movie showing abundant neurofilament movement in a neuron expressing GFP-NFM**. An example of the abundant movement that can be observed in cultured cortical neurons expressing GFP-NFM. Proximal is left and distal is right. Width of field of view: 67 μm. Time compression: 40:1 Click here for file

Additional file 2 **Movie showing abundant neurofilament movement in a neuron expressing GFP-NFM**. An example of the abundant movement that can be observed in cultured cortical neurons expressing GFP-NFM. This axon is branched, and filaments can be observed to move from the parent axon into the daughter axons and vice versa. Proximal is left and distal is right. Width of field of view: 67 μm. Time compression: 40:1 Click here for file

Additional file 3 **Movie showing neurofilament movement in a neuron expressing GFP-NFM**. A gap in the axonal neurofilament array of a cortical neuron expressing GFP-NFM. A single neurofilament moves through the gap. Proximal is left and distal is right. Width of field of view: 67 μm. Time compression: 40:1 Click here for file

Additional file 4 **Movie showing neurofilament movement in a neuron co-expressing GFP-NFM and N256S-kinesin-1A**. A gap in the axonal neurofilament array of a cortical neuron co-expressing GFP-NFM and N256S-kinesin-1A. Some jiggling movement is apparent at the edges of the gap, but no filaments move into the gap. Proximal is left and distal is right. Width of field of view: 67 μm. Time compression: 40:1 Click here for file

Additional file 5 **Movie showing neurofilament movement in a neuron co-expressing GFP-NFM and wild type kinesin-1A**. A gap in the axonal neurofilament array of a cortical neuron co-expressing GFP-NFM and wild type kinesin-1A. A neurofilament moves through the gap and then a shorter filament enters that gap. Proximal is left and distal is right. Width of field of view: 67 μm. Time compression: 40:1 Click here for file

Additional file 6 **Movie showing microtubule plus-end "comets" in a neuron expressing YFP-EB1**. Microtuble plus-end "comets" in the axon of a cortical neuron expressing YFP-EB1. Proximal is left and distal is right. Note that all the comets move anterogradely, confirming the plus-end distal orientation of the microtubules in these axons. A kymograph generated from this movie is shown in Figure 7A. Width of field of view: 42 μm. Time compression: 20:1 Click here for file
